# A conformation ensemble approach to protein residue-residue contact

**DOI:** 10.1186/1472-6807-11-38

**Published:** 2011-10-12

**Authors:** Jesse Eickholt, Zheng Wang, Jianlin Cheng

**Affiliations:** 1 Department of Computer Science, University of Missouri, Columbia, MO 65211, USA; 2 Informatics Institute, University of Missouri, Columbia, MO 65211, USA; 3 C. Bond Life Science Center, University of Missouri, Columbia, MO 65211, USA

## Abstract

**Background:**

Protein residue-residue contact prediction is important for protein model generation and model evaluation. Here we develop a conformation ensemble approach to improve residue-residue contact prediction. We collect a number of structural models stemming from a variety of methods and implementations. The various models capture slightly different conformations and contain complementary information which can be pooled together to capture recurrent, and therefore more likely, residue-residue contacts.

**Results:**

We applied our conformation ensemble approach to free modeling targets from both CASP8 and CASP9. Given a diverse ensemble of models, the method is able to achieve accuracies of. 48 for the top *L*/5 medium range contacts and. 36 for the top *L*/5 long range contacts for CASP8 targets (*L *being the target domain length). When applied to targets from CASP9, the accuracies of the top *L*/5 medium and long range contact predictions were. 34 and. 30 respectively.

**Conclusions:**

When operating on a moderately diverse ensemble of models, the conformation ensemble approach is an effective means to identify medium and long range residue-residue contacts. An immediate benefit of the method is that when tied with a scoring scheme, it can be used to successfully rank models.

## Background

Even after many years of intense attention and development, *de novo *protein structure prediction remains a difficult and open problem. In part, this is due to the inadequacy of current *de novo *sampling techniques which are incapable of guiding the folding process through such a vast conformational space [1-3]. To address this issue, several have proposed the use of long range contacts to reduce the size of the conformational search space. Studies have shown that with as few as *L*/8 long-range contacts (*L *being the sequence length) proteins can be folded and moderate resolution models generated [4,5]. Additional uses of protein residue-residue contacts include applications such as model evaluation, model selection and ranking [6-8], and drug design [9].

Given the importance and applicability of protein contacts, considerable effort has been put forth to develop methods which can predict protein residue-residue contacts. The majority of these methods can be categorized into three groups based on machine learning, templates or correlated mutations. Machine learning approaches make predictions by employing techniques such as neural networks, support vector machines or hidden Markov models trained on contacts from experimental structures [10-16]. Template based methods rely on the detection of similar structures (ie templates) by means of threading or homology and once identified, extract contacts from the templates as predictions [16-18]. Recently, more sophisticated template based approaches have been developed which attempt to combine contacts contained in differing conformations among identified templates. This is done by weighting the contacts contained within the templates based on evolutionary distance between the templates and target sequence [19]. Methods based on correlated mutation identify correlated changes in residues as evidenced in multiple sequence alignments and then exploit this information to predict residue-residue contacts [20-24]. Both machine learning and correlated mutation methods are considered *ab-initio *methods since no structural template information is used. One additional method which does not fall under the umbrella of the three categories mentioned is the extraction of contacts from 3D structural models generated for a protein. This approach has been used by the CASP assessors [25,26], a few CASP predictors such as SMEG-CCP (see CASP8 abstracts), and in scoring protein models [8].

In spite of the effort and attention that contact prediction has been given, the accuracy of long range contact predictions still remains quite low for hard targets. For these targets, accuracies typically range from 20 to 35% depending on number of contacts considered, distance thresholds and dataset [13,15,16]. Results from the eighth and ninth Critical Assessment of Techniques for Protein Structure Prediction (CASP) report that for free modeling (ie hard) targets, the average accuracy for long range contacts is routinely in the range of 20 to 25% [25,27].

Here we present a conformation ensemble approach for contact prediction. The approach is partially motivated by the view that while current protein structure predictions methods infrequently capture the overall conformation of hard targets, they do often capture portions of it. By pooling together a number of models stemming from varying alignments, templates, methods and implementations, it is possible to create an ensemble of conformations which represent portions of possible conformations for the target. The various models can capture slightly different conformations and contain complementary information which can be pooled together to capture recurrent, and therefore more likely, residue-residue contacts regardless of the particular conformation. The method works by extracting contacts from a large ensemble of possible structures generated for a protein. When evaluating the method on the CASP8 and CASP9 free modeling (FM) targets, we find that it outperforms current approaches substantially and achieves long range contact accuracies of 36% on the CASP8 FM targets and 30% on the CASP9 FM targets.

## Methods

### Datasets and Evaluation Metrics

The prediction targets used in our study were the protein domains classified as free modeling (FM) targets for CASP8 and CASP9. These are domains which did not have structural templates or the templates existed but were extremely difficult to detect [28]. For CASP8, the target domains considered were the same used in the official CASP8 assessment of contact predictors [25]. These domains included T0397 [1-82], T0405 [2-282], T0416 [124-180], T0443 [31-96], T0443 [97-118,136-173], T0460 [1-49,72-102], T0465[25-35,41-135], T0476 [2-88], T0482[5-10,19-31,35-46,49-76,96-103], T0496[4-123], T0510[236-279] and T0513[17-85]. For CASP9, we used all the domains classified as FM on the official CASP9 website (http://predictioncenter.org/casp9/domain_definitions.cgi). These domains included T0529 [7-339], T0531 [6-63], T0534 [31-80,257-384], T0534 [81-256], T0537 [65-350], T0537 [351-381], T0544 [1-135], T0547 [343-421], T0547 [554-609], T0550 [178-339], T0553 [3-65], T0553 [66-136], T0555 [12-145], T0561 [1-109,112-161], T0571 [197-331], T0578 [9-56,64-163], T0581 [27-131], T0604 [11-94], T0604 [292-496], T0608 [29-117], T0618 [6-175], T0621 [2-170], T0624 [5-73], T0629 [50-208], T0637 [1-135] and T0639 [3-126]. All the targets along with their corresponding domain definitions and experimental structures are available on the CASP websites (http://predictioncenter.org/casp8/, http://predictioncenter.org/casp9/). It should be noted that the ensemble prediction approach could be applied to hard template based modeling as well. In this study we limited ourselves to the free modeling targets as they are typically the type of target chosen when evaluating residue-residue contact prediction methods.

For the purposes of our investigation two amino acid residues are said to be in contact if the distance between their C_β _atoms (Cα for glycine) in the experimental structure is less than 8Å. Long range contacts are defined as residues in contact whose separation in the sequence is greater than or equal to 24 residues. Medium range contacts are defined by interacting residues which are 12 to 23 residues apart in the sequence. These definitions were used in accordance with previous studies [10,15,16] and CASP residue-residue contact assessments [25-27,29].

A common evaluation metric for residue-residue contact predictions is the accuracy of the top *L*/5 or *L*/10 predictions where *L *is the length of the protein in residues. If evaluating predictions over a domain, *L *can also be the length of the domain. Accuracy is defined as the number of correctly predicted residue-residue contacts divided by the total number of contact predictions considered. The recall is defined as the number of correctly predicted residue-residue contacts divided by the total number of true contacts. Additionally, we also calculated the number of contact predictions which were very close to a true contact. For this calculation, a prediction is considered correct if there is a true contact within ± δ residues for small values (ie 1 or 2) of δ.

### Conformation Ensemble Contact Prediction Procedure

**Figure 1:**
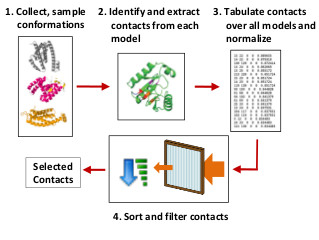
**A conformation ensemble approach for residue-residue contact prediction**. The starting point for our conformation ensemble contact predictor is a collection of structural models. From each model in the ensemble, the residue-residue contacts are extracted and then counted across all models. This list of contacts is then normalized and the most commonly occurring contacts are selected as the predicted contacts.

The starting point for our conformation ensemble contact predictor is a collection of structural models generated for a protein. This collection of structural models we define as the input ensemble. From each model in the input ensemble, the residue-residue contacts are extracted and then counted across all models. This list of contacts is then normalized so that all counts are between 0 and 1 and sorted according to frequency. At this point, the contacts can be filtered (ie restricted to a domain) and the most commonly occurring contacts are selected as the predicted contacts. The entire procedure is depicted by Figure 1.

The primary source of input ensembles was CASP. During the most recent CASP experiments, prediction groups were allowed to submit up to 5 tertiary structure predictions per target to the prediction center. The models for the groups which participated in the server category are available on the CASP website and provided us with a rich collection of ensembles for our prediction targets. On average there were 301 models in each ensemble.

## Results and Discussion

To establish an initial baseline for the effectiveness of our conformation ensemble approach, we first evaluated it on the free modeling targets from CASP8 and then tested it blindly during CASP9 as the MULTICOM human residue contact predictor. For the input ensemble, we used the tertiary structure predictions submitted by predictors in the server category. For each target domain, we calculated the precision (ie the percent of correct predictions) of top *L*/5 medium and long range contacts. This is a standard evaluation metric for contact predictors and has been used in recent CASP experiments [25-27]. As an additional evaluation metric, we calculated the precision of the top *L*/5 predictions when compared to small neighborhoods around true contacts. In this case, a prediction is counted as correct if it ± δ residues (for small δ) from a true contact. Tables 1 and 2 show the performance of the conformation ensemble method on CASP8 and CASP9 free modeling targets. The precision of the top L/5 predicted contacts on the CASP8 benchmark is 48% and 36% for medium and long range contacts respectively, and 34% and 30% on the CASP9 benchmark. If one or two residue shifting is allowed (δ = 1 or 2), the precision of the L/5 medium range contacts ranges from 55% to 77% and long range contacts from 48% to 69%.

**Table 1:** Precision and recall of conformation ensemble contact predictions on CASP8 FM targets

Evaluation criteria	Medium range contacts	Long range contacts
Top L/5	.48(.18)	.36(.08)
Top L/5, δ = 1	.70(.24)	.61(.13)
Top L/5, δ = 2	.77(.26)	.69(.14)

The performance of the conformation ensemble approach on the free modelling (FM) targets from CASP8. The input ensembles were sets of server submitted tertiary structure predictions for each FM target during CASP8. L is the sequence length of each target domain. δ is the neighbourhood size in residues. For δ = 1, a prediction is considered correct if a true contact occurs within ± 1 residues of the prediction. The precision of the predictions is shown first with the recall in parentheses.

**Table 2:** Precision and recall of conformation ensemble contact predictions on CASP9 FM targets

Evaluation criteria	Medium range contacts	Long range contacts
Top L/5	.34 (.18)	.30 (.05)
Top L/5, δ = 1	.55 (.27)	.48 (.07)
Top L/5, δ = 2	.64 (.29)	.56 (.08)

The performance of the conformation ensemble approach on the free modelling (FM) targets from CASP9. The input ensembles were sets of server submitted tertiary structure predictions for each FM target during CASP9. δ is the neighbourhood size in residues. L is the sequence length of each target domain. The precision of the predictions is shown first with the recall in parentheses.

We also compared our conformation ensemble approach with existing predictors of residue-residue contacts and to contacts extracted from individual *de novo *3D structure predictors. This assessment was conducted on the CASP9 free modeling targets. For contact predictors, we selected SVMcon [14] - a method which we developed and one of the top contact predictors in CASP9. It is worth noting that SVMcon (participating as MULTICOM-RANK server) was also among the top contact predictors in CASP8 [25]. To compare our method with contacts extracted from specific tertiary structure prediction methods, it was necessary to determine a ranking for the extracted contacts. This is because only a portion of predicted contacts are evaluated (ie, top *L*/5). To rank the contacts, we applied our ensemble approach on the 5 models submitted by BAKER-ROSSETASERVER and Zhang-Server during the CASP9 experiment. This is to say that for each predictor, we took the 5 models submitted during the CASP experiment and used these models as the input ensemble. Contacts were extracted from models and ranked according to the procedure the same procedure as that outlined by Figure 1. The results are summarized in Table 3. The results show that the precision of the ensemble approach is ≥7% higher than either a state-of-the-art sequence-based contact predictor or the contacts extracted from models generated by the top *de novo *tertiary structure predictors. This demonstrates that the ensemble-based contact prediction very likely can be used to improve *de novo *structure modeling.

**Table 3:** Comparison of contact predictors on top L/5 predictions for CASP9 FM targets

Prediction Methods	Medium range contacts	Long range contacts
Conformation ensemble	.34	.30
SVMcon	.19	.19
BAKER-ROSETTASERVER ensemble	.27	.20
Zhang-Server ensemble	.28	.23

The precision of predicted contacts obtained by various contact prediction methods. For our conformation ensemble, we used sets of server submitted tertiary structure predictions for each FM target during CASP9. SVMcon is a machine learning, sequence based contact prediction methods. BAKER-ROSETTASERVER ensemble and Zhang-Server ensemble were made by applying the conformation ensemble approach to the structural predictions made by each predictor during CASP9. L is the sequence length of each target domain.

As the quality of the contact predictions depends on the quality of the models in the ensemble, we reevaluated our method on the CASP9 targets using filtered ensembles. This allowed us to assess the method's effectiveness in coping with poor quality models and verify that the method was not relying on a small number of good models to make quality predictions. Three filtering processes were applied. In the first approach, we used ModelEvaluator [7] to predict the quality of each model and then removed those models from the ensemble whose predicted quality was below a set threshold. More specifically, we used the predicted GDT-TS value generated by ModelEvaluator and if it was below 30, the model was removed from the ensemble. We briefly mention here that GDT-TS is a standard means of assessing a model's overall quality. It is calculated by performing a superimposition of a model with the native structure and counting the number of structurally equivalent pairs of Cα atoms within given distance thresholds. Counts using distance thresholds of 1, 2, 4 and 8 Å are averaged and then normalized by the number of residues in the model [30]. This process resulted in a modest increase in prediction accuracy for long range contacts (see Table 4). In the second approach, all of the models in the starting ensemble were ranked by TM-Score [31] in comparison with the experimental structures and the top 20 scoring models were removed (see Table 4). As expected this resulted in a decrease in performance. Still, even with the best models removed from the pool, the method performs competitively with other contact prediction approaches. We should note that a few of the targets were particularly troublesome for the CASP9 predictors. For these targets, several of the top ranked models had TM-Scores in the. 20 to. 30 range and at this level the TM-Score is not an effective tool for accessing model quality. For these targets removing the top 20 scoring models may not have significantly decreased the quality of the ensemble. The third filtering approach involved creating an ensemble which consisted only of the top 20 scoring models when ranked by TM-Score in comparison with the experimental structures. The accuracy of long range contact predictions stemming from these ensembles was notably higher than that of the full, unfiltered ensembles but quite similar to the performance of the ensembles in which the poor models had been filtered out.

**Table 4:** Precision of top L/5 contact predictions obtained from filtered ensembles on CASP9 FM targets

Filter type	Medium range contacts	Long range contacts
Remove-poor	.34	.35
Remove-top	.32	.25
Only-top	.32	.37

The performance of the conformation ensemble approach when applied to filtered ensembles. The input ensembles were filtered sets of server submitted tertiary structure predictions for each FM target during CASP9. For Remove-poor, ModelEvaluator was used and any model with a predicted GDT-TS score of less than 30 was removed from an ensemble. For Remove-top, the top 20 models when ranked by TM-Score were removed from an ensemble. For Only-top, the ensemble consisted of only the top 20 models when ranked by TM-Score. L is the sequence length of each target domain.

**Figure 2:**
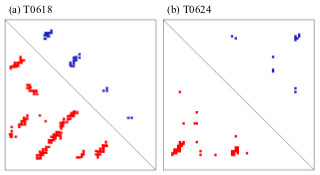
**Contact maps for CASP9 targets T0618 and T0624**. Visualized contact maps for (a) T0618 and (b) T0624. The lower portion of each figure represents true long range contacts (colored red) extracted from the experimental structure. The upper portion shows the top L/5 predicted long range contacts obtained from the conformation ensemble. The contacts cover several distinct regions of long range interaction and show their proximity to true contacts.

Given a diverse pool of models, the conformation ensemble approach performs better than existing contact prediction methods. The method is rather robust as well. Removing poor quality models or the best models from the starting ensembles does not significantly affect performance. In this work we did not directly address the usability of contacts predicted by our conformation ensemble approach to aid in tertiary structure prediction. It is a matter which we hope to explore further in a future investigation. Nevertheless, we are optimistic that the contacts will prove useful. This is due to the high accuracy of the contact predictions when evaluating them in neighborhoods of true contacts and also the clustered nature of the contacts' distribution. This is particularly true for short to medium length proteins and Figure 2 depicts results which are typical for such proteins. Their distribution and location which respect to several long range interactions indicate that they would be effective in reducing or concentrating the conformational search space which must be explored during *de novo *structure prediction.

One application of our conformational ensemble approach which we demonstrate here is its usability and effectiveness in ranking models. It should be noted that use of predicted contacts to rank and select models has been studied previously and shown to be useful [6,8]. Motivated by these efforts, we developed our own scoring scheme to rank models using contacts obtained by the conformation ensemble approach. To rank models, we used our conformational ensemble approach to generate contacts for each FM target. We then scored the models based on how well they satisfied the predicted top *L *medium range contacts and all long range contacts. More specifically, we calculated the percentage of the predicted medium range contacts satisfied exactly, the percentage of predicted medium range contacts satisfied within 1 residue (ie, δ = 1), the percentage of predicted long range contacts satisfied exactly and the percentage of predicted long range contacts satisfied within 1 residue. The sum of these percentages was calculated and used to rank the models.

One measure of the effectiveness of a ranking scheme is loss. The loss for a target is defined as the difference in GDT-TS score [30,32,33] between the best model in the group and the top ranked model. Table 5 shows the average loss per target for this simple ranking strategy based on our conformational ensemble approach along with the performances of two other ranking strategies and a random baseline measure. MULTICOM (QA) is a consensus based approach which ranks models using a combination of quality assessment (QA) values from other QA predictors. MULTICOM-CLUSTER (QA) is a pairwise model comparison approach that uses the average structural similarity between a model and all other models in the pool as its predicted quality score for model ranking. Both MULTICOM (QA) and MULTICOM-CLUSTER (QA) were among the top QA predictors in CASP9 and the former was also among the top QA predictors in CASP8 [34]. For the random baseline measure, we ranked all models by GDT-TS score and used the middlemost to calculate the loss.

**Table 5:** The average loss on CASP9 FM targets

Ranking Mechanism	Avg. Loss (in GDT-TS score)
Scoring w/conformation ensemble contacts	0.07
MULTICOM (QA)	0.07
MULTICOM-CLUSTER (QA)	0.08
Random baseline measure	0.17

Models ranked by satisfaction of contacts predicted by conformation ensemble approach. Random baseline measure is the loss of middlemost model from a group when ranked by GDT-TS score.

As indicated in Table 5, the model rankings based on contacts obtained by our conformation ensemble approach are indeed very competitive and on par with those stemming from model quality assessment programs, which performed much better than the random baseline approach. The simple scoring scheme we used to rank models rewards those models which characterize the residue-residue interactions which were most common across the ensemble. Thus, the ability to effectively rank models using contacts obtained by our conformation ensemble approach indicates that the method is able consolidate information about the protein's overall structure across the models. Here, we also note that this ranking strategy (ie, extracting contacts from models and using them as a means to rank the models) could be applicable to any protein structure prediction pipeline which produces a large number of structures in the course of making a 3D model.

**Figure 3:**
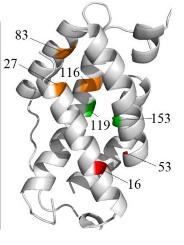
**Key long range interactions for T0618**. Several tertiary structure predictors had difficulty arranging the helical bundles for this target. Our conformation ensemble approach correctly predicted several key long range interactions for this target which help pull the helical bundles together. The input ensemble was the collection of server submitted models for T0618 during CASP9. The long range interactions are 16-53 (red), 119-153 (green) and 83-116 and 27-116 (orange).

A principle advantage of this approach is its ability to consolidate contact information across multiple models. Target T0618 is an excellent example. Several of the models submitted for target T0618 had misplaced some of the helical bundles. By pooling all of the models together into an ensemble and extracting the most common (ie, top) long range contacts, four key long range interactions can be identified (Figure 3). To check that these key contacts were not coming from a limited number of models but rather from the entire pool, we filtered the ensemble of models for this target in a variety of ways (eg, leave one predictor out, leave top 20 models out, etc). In doing so, we did not see any dependency of the key contacts to any one structural predictor or the top ranked models. For instance, if we leave out all of the models from QUARK [35] (ie, one of the most accurate *de novo *tertiary structure predictors) all four key long range interactions are still present in the predicted contacts.

To further evaluate the method's effectiveness in collecting and consolidating contacts across an ensemble of models, we clustered long range predicted contacts and then calculated the coverage of these clusters by each model in the ensemble. By doing so, we could determine if the conformation ensemble approach was pulling together more localized contacting clusters or if it was simply identifying a combination of interacting clusters which was already quite prevalent and represented in the individual models. To cluster predicted contacts, we grouped contacts based on their separation in sequence. If two contacts were within 4 residues in sequence they were placed in the same cluster. After the clusters were formed, the predicted contact closest to the average position (ie, index) in sequence to all of the contacts in a cluster was selected as the representative contact for the cluster. This list of representative contacts was filtered and only those representative contacts that were within 4 residues of a true contact were retained. Then each model was checked and the coverage of the clusters calculated. A cluster was considered covered if there was a contact in the model within 4 residues of the cluster's representative contact. Table 6 summarizes the results of this evaluation for a number of CASP9 FM targets. These results demonstrate the conformation ensemble approach is capable pooling contact data across the ensemble as the percentage of models that covers all or most of the contact clusters is low.

**Table 6:** Representation of predicted contact clusters in an ensemble

Target	Num. Clusters	Cluster Coverage (percentage of models from ensemble with stated coverage)			
T0534[31-80,257-384]	5	5(.05)	4(.14)	3(.16)	2(.17)

T0534[81-255]	3	3(.11)	2(.24)	1(.23)	0(.40)

T0544[1-135]	7	7(.07)	6(.12)	5(.13)	4(.14)

T0550[178-339]	13	11(.01)	10(.01)	9(.07)	8(.09)

T0561[1-109,112-161]	5	5(.12)	4(.12)	3(.20)	2(.16)

T0571[197-331]	6	6(.03)	5(.08)	4(.10)	3(.16)

T0608[29-117]	5	5(.06)	4(.08)	3(.11)	2(.17)

T0621[2-170]	6	6(.03)	5(.10)	4(.19)	3(.22)

Long range contacts predicted by the conformation ensemble are clustered for a number of CASP9 FM targets. The cluster coverage is the percentage of models in the ensemble that cover a given number of clusters recovered by the ensemble method. A cluster is considered covered by a model if the model contains a contact within 4 residues of the cluster's representative contact. For each target, the cluster coverages are calculated for the top four cluster counts. Num. Clusters (column 2) is the total number of true contact clusters recovered by the ensemble method for the target. Other columns followed list the percent of models in the pool containing a specific number of the true clusters. The results show that the ensemble method can recover more contact clusters even though the proportion of models in the pool having high cluster coverage is very low.

This ability to consolidate contact information across multiple models is a concept that several protein structure predictors could use as part of their own prediction pipeline. Clustering is widely used as a means to identify more probable structures from a pool of models. However, with clustering only similar models are capable of being clustered and contribute information. With the conformation ensemble approach, all models are able to contribute and help identity likely residue-residue interactions. One could easily envision an iterative approach in which a protein structure predictor could generate a diverse set of models, extract contact data and use it to generate more models. This would allow information about the conformation space to be passed from one round to the next via the likely contacts extracted from the models.

A disadvantage of the method is its dependency on a diverse ensemble of mildly accurate 3D models. In order for the approach to work, the models generated need to be able to capture at least some local portion of the overall topology of the protein. If all of the models in the ensemble are of poor quality then the method does not perform very well.

An additional consideration which must be taken into the account is the generation of the models. In practice, one would need to generate a varied ensemble of models before using the method. This could be done using a variety of protein structure prediction methods or variants of a few approaches. The time and computing resources needed to generate the models would depend on the methods used to produce the models. These decisions would affect the general practicality and usefulness of the method as a general residue-residue contact predictor. Yet, as we have demonstrated the method is applicable to ensembles of smaller sizes and still generates relatively accuracy predictions. The size of the ensemble and the sources of the models are choices which must be made when implementing a conformational ensemble predictor and inevitably affect the time needed to make contact predictions, the accuracy of those predictions and the method's ability to extract varied contact information across the models.

## Conclusions

In this work we have presented a conformation ensemble approach for predicting protein residue-residue contacts. The method draws contact data from an ensemble of models which capture slightly different conformations and contain complementary information. This information can be pooled together to capture recurrent, and therefore more likely, residue-residue contacts. We evaluated our approach on hard targets from CASP8 and CASP9 and found that it is capable of achieving state of the art performance for medium and long range residue-residue contact prediction. We have also demonstrated that the generated contact information coupled with a simple scoring scheme is capable of effectively ranking models.

## Authors' contributions

JE, JC conceived the project. JE, JC designed the experiment. JE implemented the method and carried out experiment. JE, JC, ZW analyzed the results. JE, JC wrote the manuscript. All authors read, edited and approved the manuscript.

## References

[B1] Ben-David M Noivirt-Brik O Paz A Prilusky J Sussman JL Levy Y Assessment of CASP8 structure predictions for template free targets Proteins 2009 77 Suppl 9 50 65 19774550 10.1002/prot.22591

[B2] Bradley P Misura KMS Baker D Toward High-Resolution de Novo Structure Prediction for Small Proteins Science 2005 309 1868 1871 10.1126/science.1113801 16166519

[B3] Zhang Y Progress and challenges in protein structure prediction Current Opinion in Structural Biology 2008 18 342 348 10.1016/j.sbi.2008.02.004 18436442 PMC2680823

[B4] Li W Zhang Y Skolnick J Application of sparse NMR restraints to large-scale protein structure prediction Biophys J 2004 87 1241 1248 10.1529/biophysj.104.044750 15298926 PMC1304462

[B5] Skolnick J Kolinski A Ortiz AR MONSSTER: a method for folding globular proteins with a small number of distance restraints J Mol Biol 1997 265 217 241 10.1006/jmbi.1996.0720 9020984

[B6] Miller CS Eisenberg D Using inferred residue contacts to distinguish between correct and incorrect protein models Bioinformatics 2008 24 1575 1582 10.1093/bioinformatics/btn248 18511466 PMC2638260

[B7] Wang Z Tegge AN Cheng J Evaluating the absolute quality of a single protein model using structural features and support vector machines Proteins 2009 75 638 647 10.1002/prot.22275 19004001

[B8] Tress ML Valencia A Predicted residue-residue contacts can help the scoring of 3D models Proteins 2010 78 1980 1991 20408174 10.1002/prot.22714

[B9] Kliger Y Levy O Oren A Ashkenazy H Tiran Z Novik A Rosenberg A Amir A Wool A Toporik A Peptides modulating conformational changes in secreted chaperones: from in silico design to preclinical proof of concept Proc Natl Acad Sci USA 2009 106 13797 13801 10.1073/pnas.0906514106 19666568 PMC2728974

[B10] Bjorkholm P Daniluk P Kryshtafovych A Fidelis K Andersson R Hvidsten TR Using multi-data hidden Markov models trained on local neighborhoods of protein structure to predict residue-residue contacts Bioinformatics 2009 25 1264 1270 10.1093/bioinformatics/btp149 19289446 PMC2677742

[B11] Pollastri G Baldi P Prediction of contact maps by GIOHMMs and recurrent neural networks using lateral propagation from all four cardinal corners Bioinformatics 2002 18 Suppl 1 S62 70 10.1093/bioinformatics/18.suppl_1.S62 12169532

[B12] Xue B Faraggi E Zhou Y Predicting residue-residue contact maps by a two-layer, integrated neural-network method Proteins 2009 76 176 183 10.1002/prot.22329 19137600 PMC2716487

[B13] Tegge AN Wang Z Eickholt J Cheng J NNcon: improved protein contact map prediction using 2D-recursive neural networks Nucleic Acids Res 2009 37 W515 518 10.1093/nar/gkp305 19420062 PMC2703959

[B14] Cheng J Baldi P Improved residue contact prediction using support vector machines and a large feature set BMC Bioinformatics 2007 8 113 10.1186/1471-2105-8-113 17407573 PMC1852326

[B15] Vullo A Walsh I Pollastri G A two-stage approach for improved prediction of residue contact maps BMC Bioinformatics 2006 7 180 10.1186/1471-2105-7-180 16573808 PMC1484494

[B16] Wu S Zhang Y A comprehensive assessment of sequence-based and template-based methods for protein contact prediction Bioinformatics 2008 24 924 931 10.1093/bioinformatics/btn069 18296462 PMC2648832

[B17] Misura KM Chivian D Rohl CA Kim DE Baker D Physically realistic homology models built with ROSETTA can be more accurate than their templates Proc Natl Acad Sci USA 2006 103 5361 5366 10.1073/pnas.0509355103 16567638 PMC1459360

[B18] Skolnick J Kihara D Zhang Y Development and large scale benchmark testing of the PROSPECTOR_3 threading algorithm Proteins 2004 56 502 518 10.1002/prot.20106 15229883

[B19] Ashkenazy H Unger R Kliger Y Hidden conformations in protein structures Bioinformatics 2011 27 1941 1947 10.1093/bioinformatics/btr292 21586517

[B20] Fodor AA Aldrich RW Influence of conservation on calculations of amino acid covariance in multiple sequence alignments Proteins 2004 56 211 221 10.1002/prot.20098 15211506

[B21] Gobel U Sander C Schneider R Valencia A Correlated mutations and residue contacts in proteins Proteins 1994 18 309 317 10.1002/prot.340180402 8208723

[B22] Kundrotas PJ Alexov EG Predicting residue contacts using pragmatic correlated mutations method: reducing the false positives BMC Bioinformatics 2006 7 503 10.1186/1471-2105-7-503 17109752 PMC1654194

[B23] Olmea O Valencia A Improving contact predictions by the combination of correlated mutations and other sources of sequence information Fold Des 1997 2 S25 32 9218963 10.1016/s1359-0278(97)00060-6

[B24] Vicatos S Reddy BV Kaznessis Y Prediction of distant residue contacts with the use of evolutionary information Proteins 2005 58 935 949 10.1002/prot.20370 15645442

[B25] Ezkurdia I Grana O Izarzugaza JM Tress ML Assessment of domain boundary predictions and the prediction of intramolecular contacts in CASP8 Proteins 2009 77 Suppl 9 196 209 19714769 10.1002/prot.22554

[B26] Izarzugaza JM Grana O Tress ML Valencia A Clarke ND Assessment of intramolecular contact predictions for CASP7 Proteins 2007 69 Suppl 8 152 158 17671976 10.1002/prot.21637

[B27] Monastyrskyy B Fidelis K Tramontano A Kryshtafovych A Evaluation of residue-residue contact predictions in CASP9 Proteins 2011 10.1002/prot.23160 PMC3226919 21928322

[B28] Tress ML Ezkurdia I Richardson JS Target domain definition and classification in CASP8 Proteins 2009 77 Suppl 9 10 17 19603487 10.1002/prot.22497 PMC2805415

[B29] Grana O Baker D MacCallum RM Meiler J Punta M Rost B Tress ML Valencia A CASP6 assessment of contact prediction Proteins 2005 61 Suppl 7 214 224 16187364 10.1002/prot.20739

[B30] Zemla A Venclovas Moult J Fidelis K Processing and evaluation of predictions in CASP4 Proteins 2001 Suppl 5 13 21 10.1002/prot.10052 11835478

[B31] Zhang Y Skolnick J Scoring function for automated assessment of protein structure template quality Proteins 2004 57 702 710 10.1002/prot.20264 15476259

[B32] Zemla A LGA: A method for finding 3D similarities in protein structures Nucleic Acids Res 2003 31 3370 3374 10.1093/nar/gkg571 12824330 PMC168977

[B33] Zemla A Venclovas C Moult J Fidelis K Processing and analysis of CASP3 protein structure predictions Proteins 1999 Suppl 3 22 29 10.1002/(sici)1097-0134(1999)37:3+<22::aid-prot5>3.3.co;2-n 10526349

[B34] Cozzetto D Kryshtafovych A Tramontano A Evaluation of CASP8 model quality predictions Proteins 2009 77 Suppl 9 157 166 19714774 10.1002/prot.22534

[B35] Xu D Zhang J Roy A Zhang Y Automated protein structure modeling in CASP9 by I-TASSER pipeline combined with QUARK-based *ab initio *folding and FG-MD-based structure refinement Proteins 2011 10.1002/prot.23111 PMC3228277 22069036

